# The Role of the Collateral Circulation in Stable Angina: An Invasive Placebo-Controlled Study

**DOI:** 10.1161/CIRCULATIONAHA.125.074687

**Published:** 2025-10-27

**Authors:** Christopher A. Rajkumar, Michael J. Foley, Fiyyaz Ahmed-Jushuf, Shayna Chotai, Florentina A. Simader, Muhammad Mohsin, Ahmed Salih, Sashiananthan Ganesananthan, Nina Bual, Ricardo Petraco, Sukhjinder S. Nijjer, Sayan Sen, Joban Sehmi, Neil Ruparelia, Jason N. Dungu, Alamgir Kabir, Kare Tang, Reto Gamma, John R. Davies, Tushar Kotecha, Graham D. Cole, James P. Howard, Thomas R. Keeble, Gerald J. Clesham, Peter D. O’Kane, Frank E. Harrell, Darrel P. Francis, Matthew J. Shun-Shin, Rasha K. Al-Lamee

**Affiliations:** 1 National Heart and Lung Institute, Imperial College London, London, UK (C.A.R., M.J.F., F.A.-J., S.C., F.A.S., M.M., A.S., S.G., R.P., G.D.C., J.P.H., D.P.F., M.J.S.-S., R.K.A.-L.).; 2 Imperial College Healthcare NHS Trust, London, UK (C.A.R., M.J.F., F.A.-J., S.C., F.A.S., S.G., N.B., R.P., S.S.N., N.R., G.D.C., J.P.H., D.P.F., M.J.S.-S., R.K.A.-L.).; 3 One Heart Clinic, London, UK (S.S.).; 4 West Hertfordshire Hospitals NHS Trust, Watford, UK (J.S.).; 5 Royal Berkshire NHS Foundation Trust, Reading, UK (N.R.).; 6 Essex Cardiothoracic Centre, Mid and South Essex NHS Foundation Trust, Basildon, UK (J.N.D., A.K., K.T., R.G., J.R.D., T.R.K., G.J.C.).; 7 Anglia Ruskin School of Medicine and MTRC, Chelmsford, UK (J.N.D., T.R.K., G.J.C.).; 8 Royal Free London NHS Foundation Trust, London, UK (T.K.).; 9 University Hospitals Dorset NHS Foundation Trust, Bournemouth, UK (P.D.O.).; 10 Vanderbilt University School of Medicine, Nashville, TN (F.E.H.).

**Keywords:** collateral circulation, coronary artery disease, stable angina

## Abstract

**BACKGROUND:**

Little correlation exists between the burden of ischemia and severity of angina in patients with stable coronary artery disease. This placebo-controlled, n-of-1 study investigated the relationship between ischemia, the collateral circulation, and symptoms in stable coronary artery disease. Additionally, it explored the association between progressive collateral recruitment and ischemic preconditioning.

**METHODS:**

Fifty-one participants with severe single-vessel coronary artery disease and angina were recruited. Antianginal medications were stopped, and daily angina symptoms were documented using a dedicated smartphone application (ORBITA [Objective Randomized Blinded Investigation With Optimal Medical Therapy of Angioplasty in Stable Angina] app) for 14 days before undergoing invasive pressure wire studies and coronary flow reserve assessment. Each participant then underwent four 60-s episodes of low-pressure balloon occlusion across their coronary stenosis. Each episode was paired with an audiovisually identical placebo inflation in a randomized order. After each episode, participants scored pain intensity on a 10-point scale, and a placebo-controlled pain intensity score was calculated. Collateral flow index was calculated from simultaneous measures of aortic, right atrial, and distal coronary wedge pressure during balloon occlusion. Higher *Pr* values from Bayesian models indicate a greater likelihood of association.

**RESULTS:**

The mean (±SD) age of participants was 63±9 years, and 78% were men. The median (interquartile range) fractional flow reserve was 0.68 (0.57–0.79), the median instantaneous wave-free ratio was 0.80 (0.48–0.89), and the median coronary flow reserve was 1.42 (1.08–1.85). Daily angina frequency showed little correlation with severity of ischemia, as assessed by fractional flow reserve (Somers’ D 0.124, *Pr*=0.057) or instantaneous wave-free ratio (Somers’ D 0.056, *Pr*=0.150). However, there was strong evidence of an association between lower fractional flow reserve and instantaneous wave-free ratio values and greater collateral flow (Somers’ D 0.302, *Pr*=0.998 and Somers’ D 0.316, *Pr*=0.999, respectively). There was also strong evidence of an association between more collateralization (higher collateral flow index) and lower pain intensity scores (Somers’ D 0.341, *Pr*=0.999). Finally, pain intensity scores and collateral flow index remained stable between sequential balloon occlusion episodes within individual patients, indicating little evidence of ischemic preconditioning.

**CONCLUSIONS:**

Coronary collateralization is associated with ischemic burden and may reduce the intensity of ischemic chest pain. This may explain the nonlinear relationship between stenosis, ischemia, and angina.

**REGISTRATION:**

URL: https://www.clinicaltrials.gov; Unique identifier: NCT04280575.

**Clinical Perspective**

**What Is New?**

- In patients with stable single-vessel coronary artery disease, the severity of daily symptoms correlates poorly with the burden of myocardial ischemia.
- As the burden of myocardial ischemia increases, there is a corresponding rise in coronary collateralizing pressures within the recipient vessel.
- Coronary collateralization is associated with reduced ischemic chest pain, demonstrated for the first time under placebo-controlled conditions.

**What Are the Clinical Implications?**

- Symptom severity alone may not accurately reflect the burden of ischemia in patients with stable coronary artery disease.
- Enhanced coronary collateralization in response to increased ischemic burden may reduce symptom severity through improved myocardial perfusion.
- The relationship between ischemia and angina is influenced by multiple patient-specific factors, which may limit the predictability of symptom relief after revascularization.

Angina attributable to obstructive disease in the epicardial coronary arteries is often regarded as a straightforward mechanistic construct. Conventional teaching describes restricted augmentation of blood flow through a stenosis resulting in ischemia, an oxygen supply-demand imbalance, which manifests as angina. Intuitively, one might expect that the most severe stenoses, or the largest burden of ischemia, would cause the most limiting symptoms. However, surprisingly, evidence reveals very little correlation between the extent of ischemia and severity of angina.^1–4^ Despite the clinical implications of this disconnect, the underlying causes are not well understood. Consequently, guidelines that rely upon anatomic or physiological measures of stenosis severity to guide revascularization may fail to identify patients with the most to gain.^5,6^

One possible explanation may lie in the coronary collateral circulation, which provides an alternative source of blood flow through vascular adaptation and neoangiogenesis. Anastomoses between the left and right coronary arteries were first described in autopsy samples by Richard Lower in 1669.^7^ Although their functional significance was not appreciated at the time, William Heberden’s initial account of angina pectoris described a patient who was cured of all symptoms by sawing wood for half an hour per day.^8^ This intriguing phenomenon, in which angina diminished with repeated effort, was later used by physicians who instructed patients to “walk through the pain,” a hypothesis founded upon recruitment and maturation of coronary collaterals.^9^

In some clinical settings, the collateral circulation is recognized to have clear functional relevance. In chronic total occlusions, a mature collateral circulation can maintain viability of the subtended myocardium.^10^ Similarly, in acute myocardial infarction, the presence of angiographic collaterals limits infarct size and is associated with improved clinical outcomes.^11^ Beyond the coronary circulation, similar phenomena are described within the peripheral and cerebral circulations.^12,13^ However, little is known about the extent or functional relevance of the collateral circulation in modulating the symptoms of stable angina.

An invasive, placebo-controlled, n-of-1 study was performed to answer 3 important questions. First, in patients with stable coronary artery disease (CAD), does more severe ischemia result in more frequent day-to-day angina? Second, in patients with stable angina, is more ischemia associated with more collateralization? Finally, does the development of a collateral circulation reduce the severity of ischemic chest pain?

## Methods

The data, analytical methods, and study materials will not be made available to other researchers for the purposes of reproducing the results or replicating the procedure.

### Study Design and Participants

The multicenter, n-of-1, placebo-controlled ORBITA-STAR (Objective Randomized Blinded Investigation With Optimal Medical Therapy of Angioplasty in Stable Angina-Systematic Trial of Angina Assessment Prior to Revascularization)^14^ was approved by the London central research ethics committee (reference 19/LO/1194). All participants provided written informed consent.

The study recruited patients with symptoms of angina or angina-equivalent symptoms with severe single-vessel CAD (≥70%) on invasive coronary angiography or computed tomography coronary angiography who were referred for revascularization. All inclusion and exclusion criteria are listed in Tables S1 and S2.

### Study Protocol

**Figure 1.**
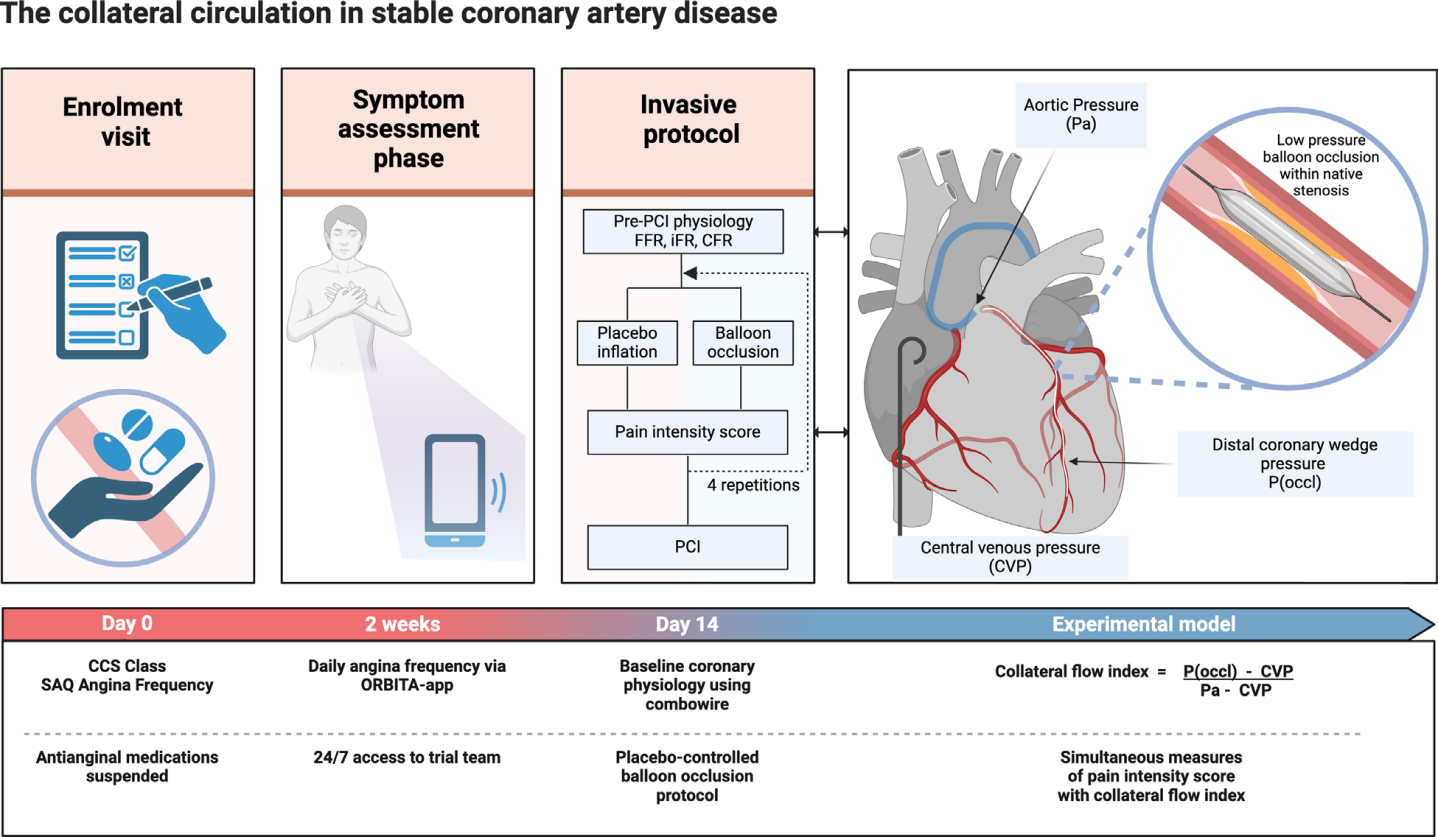
**Study design and experimental model for the placebo-controlled balloon occlusion protocol.** A guiding catheter was placed into the coronary ostium. A pressure and Doppler sensor–tipped Combowire was placed beyond the stenosis. A pigtail catheter was placed into the right atrium. The collateral flow index was calculated during low-pressure balloon occlusion episodes from simultaneous measures of aortic pressure (Pa) from the guiding catheter, distal coronary wedge pressure (P(occl)) from the Combowire distal to the occluding balloon, and central venous pressure (CVP) from the pigtail catheter in the right atrium. After each episode, balloon occlusion or placebo inflation, patients reported the intensity of any pain experienced on a 10-point ordinal scale, on which 0 is no perceived sensation and 10 is the worst pain imaginable. CCS indicates Canadian Cardiovascular Society; CFR, coronary flow reserve; FFR, fractional flow reserve; iFR, instantaneous wave-free ratio; PCI, percutaneous coronary intervention; and SAQ, Seattle Angina Questionnaire.

The full methods have been previously described.^14^ The study design and experimental model are shown in Figure 1.

At enrollment, all antianginal medications were stopped. Exemptions were permitted for patients who required a beta-blocker for atrial fibrillation, left ventricular impairment, or after myocardial infarction. If patients had a diagnosis of hypertension, then any antianginal medications with antihypertensive properties were replaced with antihypertensives with no antianginal properties.

### Symptom Assessment

Symptom severity at baseline was documented using the Seattle Angina Questionnaire (SAQ).

After enrollment, patients entered a 2-week pre–percutaneous coronary intervention (PCI) symptom assessment phase. During this period, they reported their angina frequency daily using a dedicated smartphone application (ORBITA app; Figure S1). Daily reports were transmitted instantly, and all data were stored on an approved server. Patients had 24-hour/7-day access to the trial team during this period and could restart antianginal medications in collaboration with the trial team if these were required.

Before the invasive protocol, patients attended the pre-PCI assessment visit. This included repeat symptom questionnaires and dobutamine stress echocardiography.

### Invasive Protocol

Dual antiplatelet therapy was administered before the invasive protocol. Coronary angiography was performed using the radial or femoral approach. Central venous access was obtained through the femoral or brachial vein.

The invasive protocol consisted of the following 4 stages.

#### Pre-PCI Coronary Physiology

A Doppler and pressure sensor–tipped wire (Combowire; Volcano Corp, San Diego, CA) was used for all physiological assessments. Intracoronary nitrates were administered before equalization of the Combowire. The wire was then positioned distal to the coronary stenosis. Baseline fractional flow reserve (FFR), instantaneous wave-free ratio (iFR), and coronary flow reserve were measured. For hyperemic indices, an intravenous infusion of adenosine at 140 μg·kg·min was administered.

#### Collateral Flow Index and Pain Intensity Assessment During Placebo-Controlled Balloon Occlusion

The invasive protocol was designed to permit simultaneous assessments of the patient’s collateral circulation and their symptomatic response to ischemia. A diagram of the experimental model is shown in Figure 1.

A pigtail catheter was placed in the right atrium for central venous pressure monitoring. An angioplasty wire was placed alongside the Combowire, distal to the coronary stenosis. The placebo-controlled balloon occlusion protocol consisted of 4 pairs of episodes. Patients were informed when each episode started and finished. Within each pair, there was one 60-s episode of low-pressure balloon occlusion across the patient’s stenosis, designed to provoke ischemia. This was paired with a 60-s placebo inflation in which ischemia was not induced. These real and placebo episodes were performed in a randomized order (resulting in a total of 8 60-s episodes). Patients were blinded to the nature of each episode, real balloon occlusion or placebo inflation, with use of surgical drapes to ensure that the patient was not able to see the operator or the catheter laboratory screens.

##### Balloon Occlusion Episodes

A semicompliant angioplasty balloon, sized from angiographic appearances at a 1:1 balloon:artery ratio, was placed within the coronary stenosis. At the start of the episode, it was inflated to the lowest pressure that occluded flow to the distal vessel (typically 1–3 atmospheres). An injection of contrast and loss of the distal Doppler flow trace from the Combowire confirmed that the balloon was occlusive. After 60 s, the balloon was deflated and withdrawn from the stenosis into the guiding catheter.

##### Placebo Inflation

The deflated angioplasty balloon remained within the guiding catheter. To preserve blinding, the operator used a decoy balloon outside the patient’s body, inflating it and deflating it at the start and end of the episode, respectively, to recreate the sound of the balloon occlusion episode. This ensured that placebo episodes were audiovisually identical to the balloon occlusion episodes.

### Pain Intensity Scoring

Immediately after termination of each episode, patients reported the intensity of the pain experienced on an ordinal scale from 0 (no perceived sensation) to 10 (worst pain imaginable). Ascertainment of the pain intensity score was carried out in an identical manner regardless of whether the episode was real balloon occlusion or placebo.

After each episode, balloon occlusion or placebo inflation, a recovery period with a minimum duration of 30 s was mandatory before the next episode could begin. If symptoms or ECG abnormalities persisted, then the recovery time was extended until they had resolved, plus an additional 30 s to avoid carryover to the next episode. The Doppler flow trace from the Combowire distal to the stenosis was used to confirm that hyperemia, induced by balloon occlusion, had resolved and that coronary flow had returned to baseline before the next episode began.

### Calculation of the Collateral Flow Index

**Figure 2.**
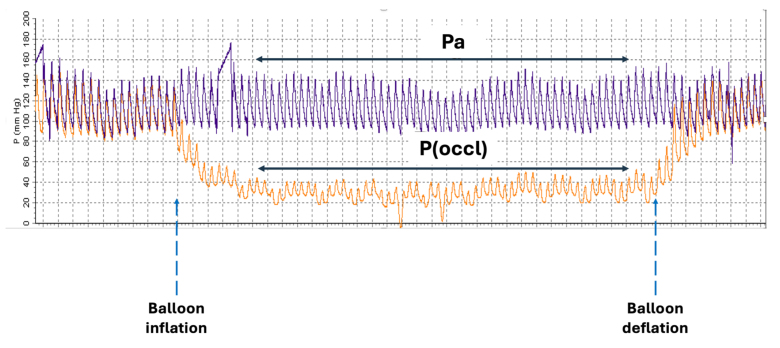
**Example pressure waveforms displaying simultaneous aortic pressure and distal coronary wedge pressure during a 60-s balloon occlusion episode.** The distal coronary pressure (green line) can be seen to fall to a steady pulsatile nadir as the occluding balloon is inflated (distal coronary wedge pressure). This pressure is a surrogate of collateral blood flow. As the occluding balloon is deflated, the distal coronary pressure returns to baseline. Pa indicates aortic pressure and P(occl), distal coronary wedge pressure.

The collateral flow index (CFI) is a validated invasive metric of hemodynamic collateral perfusion pressures.^15,16^ During each balloon occlusion episode, the pressure-derived CFI was calculated according to published methods, using hemodynamic data from the final 10 s of each 60-s balloon occlusion episode.^15,16^ This required simultaneous measurements of aortic pressure, distal coronary wedge pressure from the Combowire positioned distal to the occluding balloon, and finally the central venous pressure, measured from the pigtail catheter in the right atrium. Example pressure waveforms are shown in Figure 2.

The CFI was calculated using the following formula:


$$\begin{array}{l} CFI=\frac{P\left(occl\right)-CVP}{Pa-CVP} \end{array}$$

where *P(occl*) is the distal coronary wedge pressure during balloon occlusion, *Pa* is the aortic pressure, and *CVP* is the central venous pressure. The CFI typically ranges from 0 to 1 (no units), where higher values indicate greater collateral flow. Negative values are possible when the central venous pressure is greater than the distal coronary wedge pressure.

#### PCI of the Target Lesion

After the placebo-controlled balloon occlusion protocol, all participants underwent PCI of the target lesion with implantation of drug-eluting stents. Use of drug-coated balloons was permitted when clinically indicated. Postdilation of stents and use of intracoronary imaging was recommended.

#### Post-PCI Coronary Physiology

After PCI, intracoronary nitrates were administered, and the Combowire was used to reassess post-PCI FFR, iFR, and coronary flow reserve.

### Outcomes

The placebo-controlled pain intensity score was calculated on an individual basis as the mean of the pain intensity during balloon occlusion episodes after subtraction of the mean pain intensity during the placebo inflation episodes.

The stress echocardiography score was double-reported by 3 imaging consultants (J.S., G.D.C., and D.P.F.) using an online reporting tool and previously reported methodology.^17^ Reporters were blinded to the coronary anatomy, physiological parameters, and placebo-controlled pain intensity scores.

### Statistical Analysis

The associations between the physiological parameters (FFR and iFR) and stress echocardiography score with symptom parameters were assessed using a Bayesian ordinal proportional odds model. Nonlinearity in the predictor was allowed with use of a restricted cubic spline with 3 knots. For the intercepts, a Dirichlet distribution with package default concentration parameters was used as the prior. For other covariates, a virtually flat prior was used, with a mean of 0 and an SD of 100. This model produces an odds ratio that was transformed to the original scale using a weighted mean of the possible response levels with weights equal to the cell probabilities from the proportional odds model. The strength of association was measured using Somers’ D, an ordinal equivalent of a correlation coefficient, ranging from 0 to 1, with 0 equivalent to no correlation and 1 being a perfect fit. The difference in symptoms between a patient at the 25th and 75th centile of severity was calculated in addition to the associated probability of difference. The coefficients associated 95% credible intervals, and the probability that these exceeded 0 are presented.

To assess the impact of repeated balloon inflations (ie, testing for evidence of preconditioning), the inflation number (random intercept) clustered under the study ID was used as the predictor in a Bayesian ordinal proportional odds model. The probability that the inflation number coefficient was >1 is presented along with the model-predicted means on the first and last balloon inflation.

All analyses were conducted using the statistical environment R (version 4.5.0) using the package “rsmb” (version 1.1-1) for Bayesian modelling.^18^

This study is registered with Clinical trials.gov (URL: https://www.clinicaltrials.gov; Unique identifier: NCT04280575).

## Results

### Patients

Fifty-one participants completed the study protocol at 5 trial centers from December 9, 2019, to May 1, 2023. The consort diagram is shown in Figure S2. There was no loss to follow-up.

Baseline characteristics are presented in Table 1. The majority (78%) of participants were men, 28% had diabetes, and 10% had undergone previous PCI. The median duration of angina at enrollment was 43 (interquartile range, 26–78) weeks. Most patients (88%) had Canadian Cardiovascular Society class II or III angina.

**Table 1.**
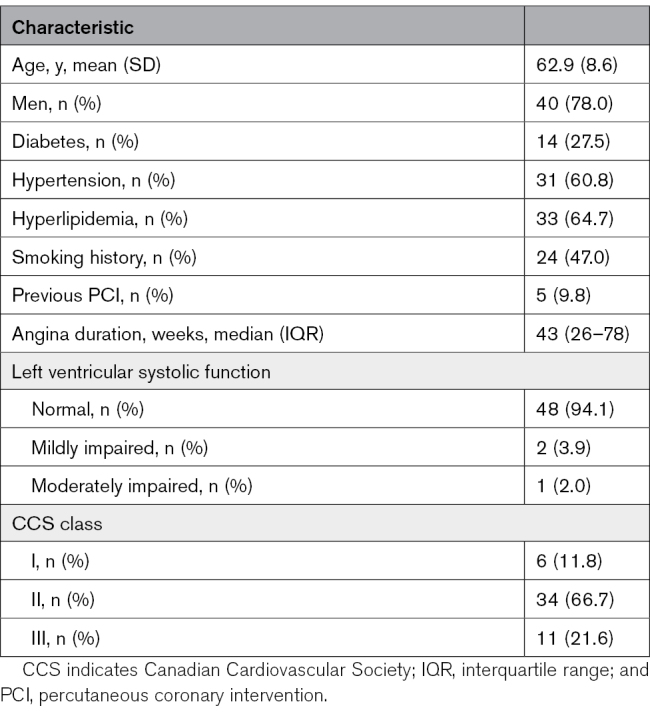
Baseline Characteristics

Anatomic and physiological characteristics of the target lesions are provided in Table 2. The target lesion was located in the left anterior descending artery in 72.5% of participants. The median diameter of stenosis by quantitative coronary angiography was 68.7% (60.0–76.4). The median pre-PCI FFR, iFR, and coronary flow reserve values were 0.68 (0.57–0.79), 0.80 (0.48–0.89), and 1.42 (1.08–1.85), respectively.

**Table 2.**
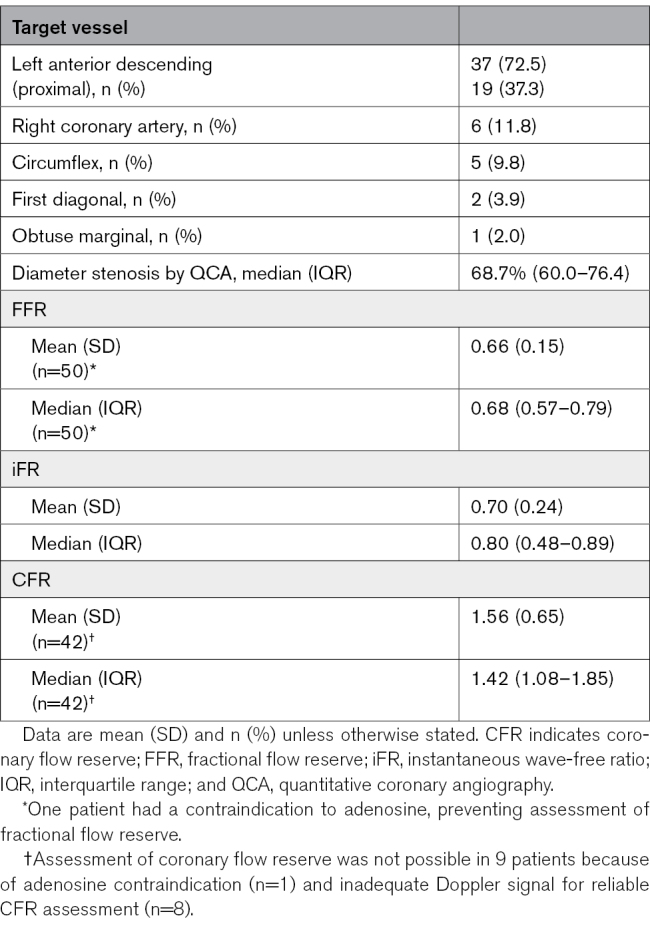
Stenosis Characteristics

### Association Between Burden of Ischemia and Daily Angina Frequency

**Figure 3.**
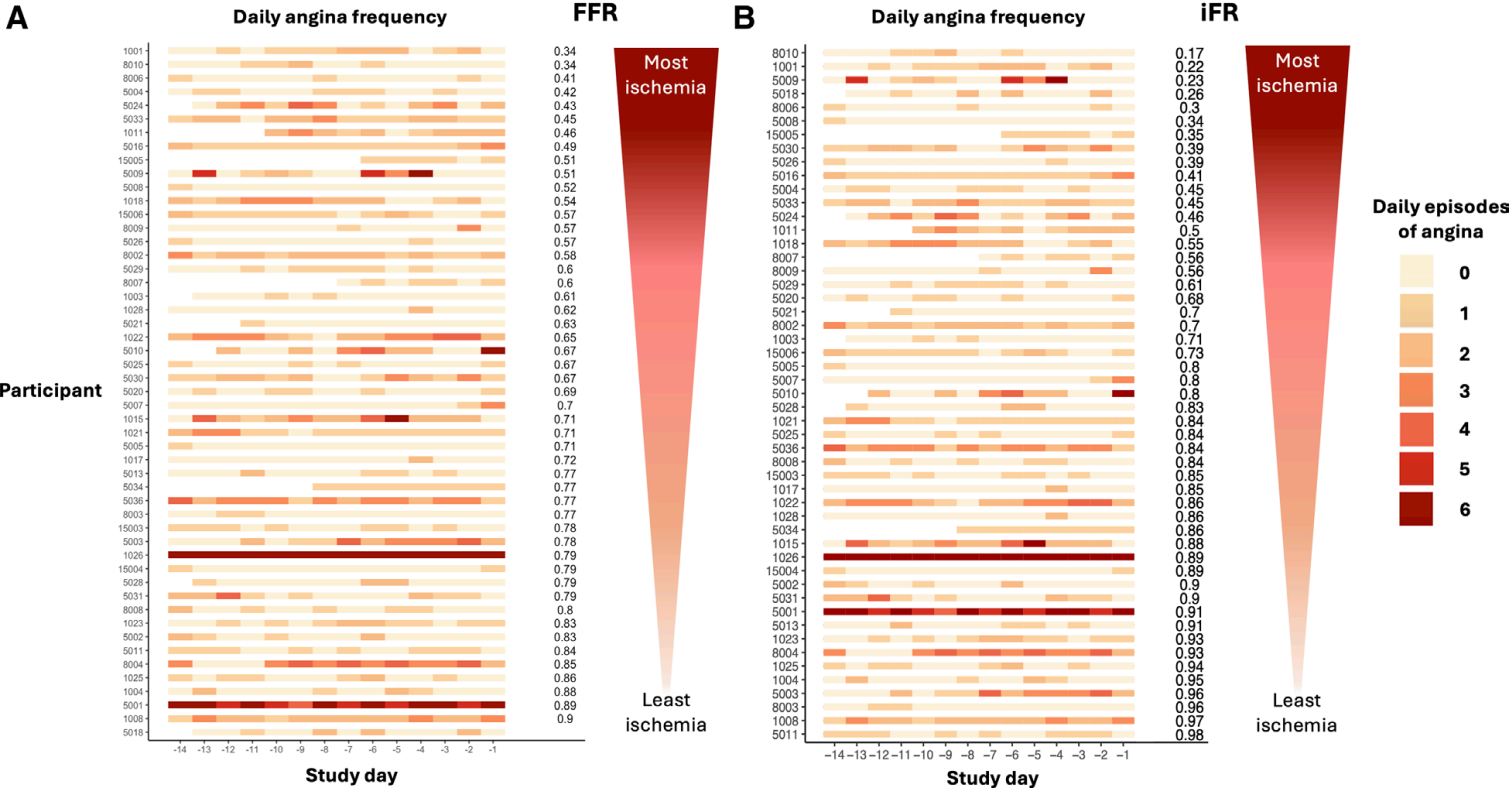
**Association between daily angina frequency reported via the smartphone app and physiological stenosis severity as assessed by FFR (A) and iFR (B).** Each patient is depicted as an individual row. Each colored panel is an individual day of the trial in which darker colors indicate greater angina frequency. The patients have been sorted with the most ischemic (lowest fractional flow reserve [FFR] and instantaneous wave-free ratio [iFR]) stenoses toward the top and the least ischemic (highest FFR and iFR) stenoses toward the bottom. To the right of each row is the absolute FFR or iFR value for each patient (blank where not measured). There was no detectable relationship between physiological stenosis severity and daily angina frequency.

Angina frequency for each patient in the 2 weeks before PCI was calculated from daily symptom reports from the ORBITA app. There was little evidence of association between severity of ischemia as assessed by FFR and mean daily angina frequency; patients with more ischemic FFR values did not have more frequent angina (Somers’ D 0.122, *Pr*>0=0.057). Similarly, there was little evidence of an association between severity of ischemia as assessed by iFR and mean daily angina frequency; patients with lower iFR values did not have more frequent angina (Somers’ D 0.052, *Pr*>0=0.150; Table 3). Individual patient data are presented in Figure 3.

**Table 3.**
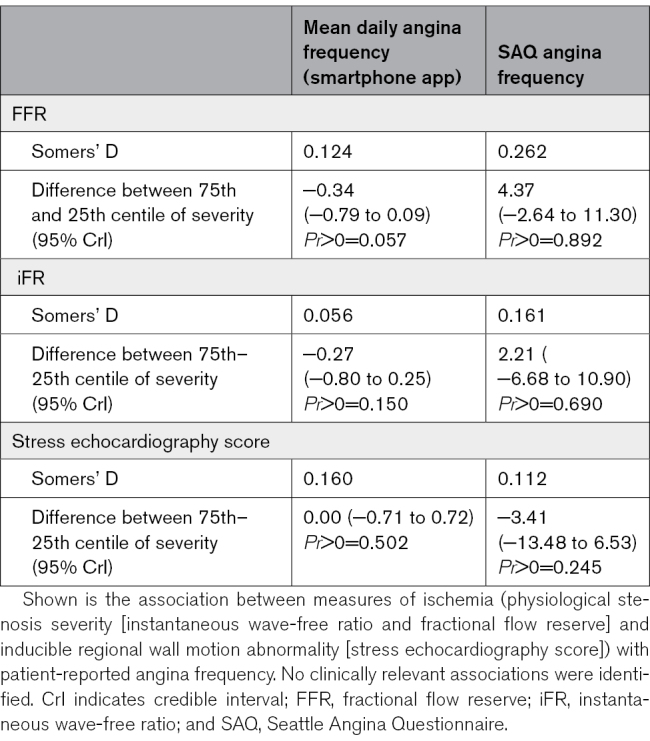
Association Between Ischemic Burden and Patient-Reported Symptoms

A weak correlation was observed between FFR and pre-PCI SAQ angina frequency, with limited evidence that patients with more ischemia had greater SAQ angina frequency (Somers’ D 0.262, *Pr*>0=0.892). There was little evidence of an association between iFR and pre-PCI SAQ angina frequency, indicating that patients with lower iFR values did not have greater SAQ angina frequency (Somers’ D 0.161, *Pr*>0=0.690).

Ischemia assessed by stress echocardiography score showed little association with either mean daily angina frequency (Somers’ D 0.160) or SAQ angina frequency (Somers’ D 0.112); more ischemia on stress echocardiography score did not predict greater angina frequency by either measure (*Pr*>0=0.502 and 0.245, respectively).

Overall, there was no evidence of a clinically discriminative value of any measure of ischemia for the burden of patient-reported symptoms (Table 3).

### Association Between Ischemia and the Collateral Circulation in Patients with Stable Angina

The collateral circulation was estimated using the mean CFI in the final 10 s of each of the 4 balloon occlusion episodes.

**Figure 4.**
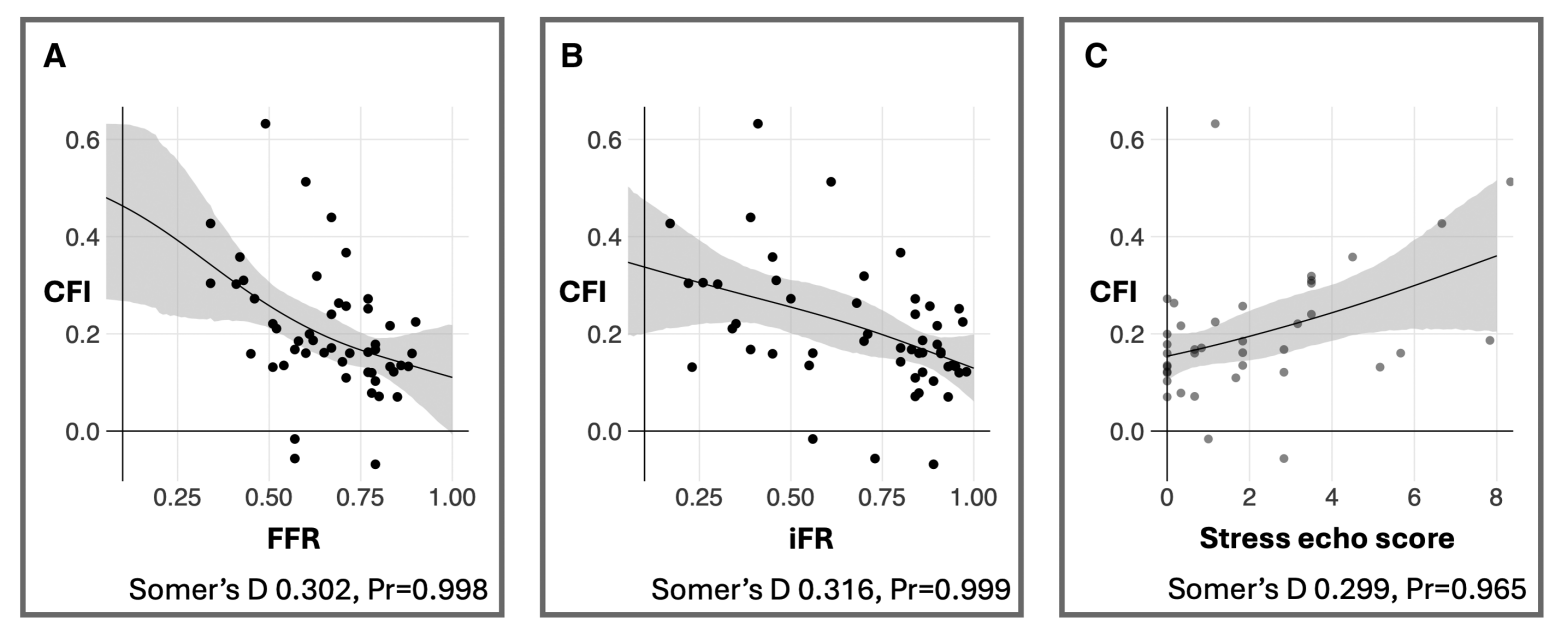
Association between the collateral flow index (CFI) and fractional flow reserve (FFR; A), the instantaneous wave-free ratio (iFR; B), and stress echocardiography score (where higher scores indicate a greater burden of ischemia; C).

There was strong evidence of an association between ischemia as assessed by FFR and collateral blood flow; lower (more ischemic) FFR values were associated with a higher CFI (Somers’ D 0.302, *Pr*>0=0.998; Figure 4A).

Similarly, there was strong evidence of association between degree of ischemia as assessed by iFR and extent of collateralization as assessed by CFI (Somers’ D 0.316, *Pr*>0=0.999; Figure 4B).

Finally, there was strong evidence of an association between noninvasive ischemia assessment with stress echocardiography score and the extent of collateralization; more ischemia on stress echocardiography predicted a higher CFI (Somers’ D 0.299, *Pr*>0=0.965; Figure 4C; Table 4).

**Table 4.**
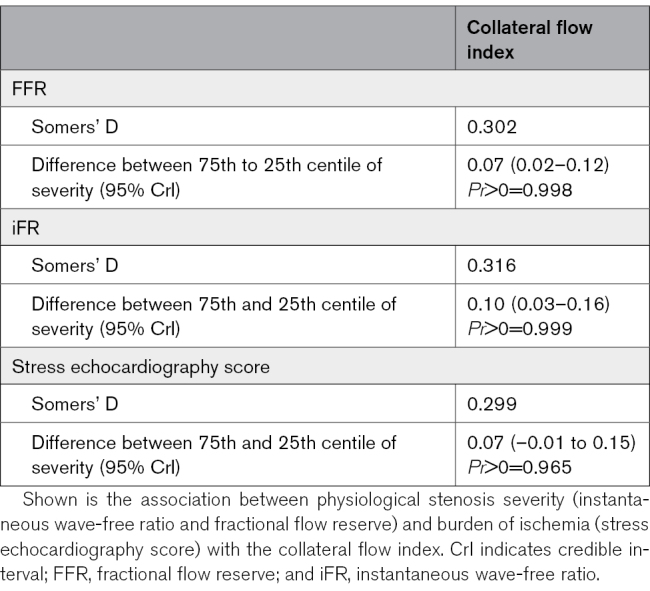
Association between Burden of Ischemia and Collateral Flow

### Association Between CFI and Pain Intensity Scores

The placebo-controlled pain intensity score was calculated for each pair of episodes by subtracting the pain score during placebo inflation from that during balloon occlusion and then taking the mean of 4 pairs for each participant. Across study participants, the observed placebo-controlled pain intensity scores ranged from 0.0 (a participant with no perceived difference in pain intensity between real and placebo episodes) to 8.5 (the participant with the maximal difference in pain intensity between real and placebo episodes).

**Figure 5.**
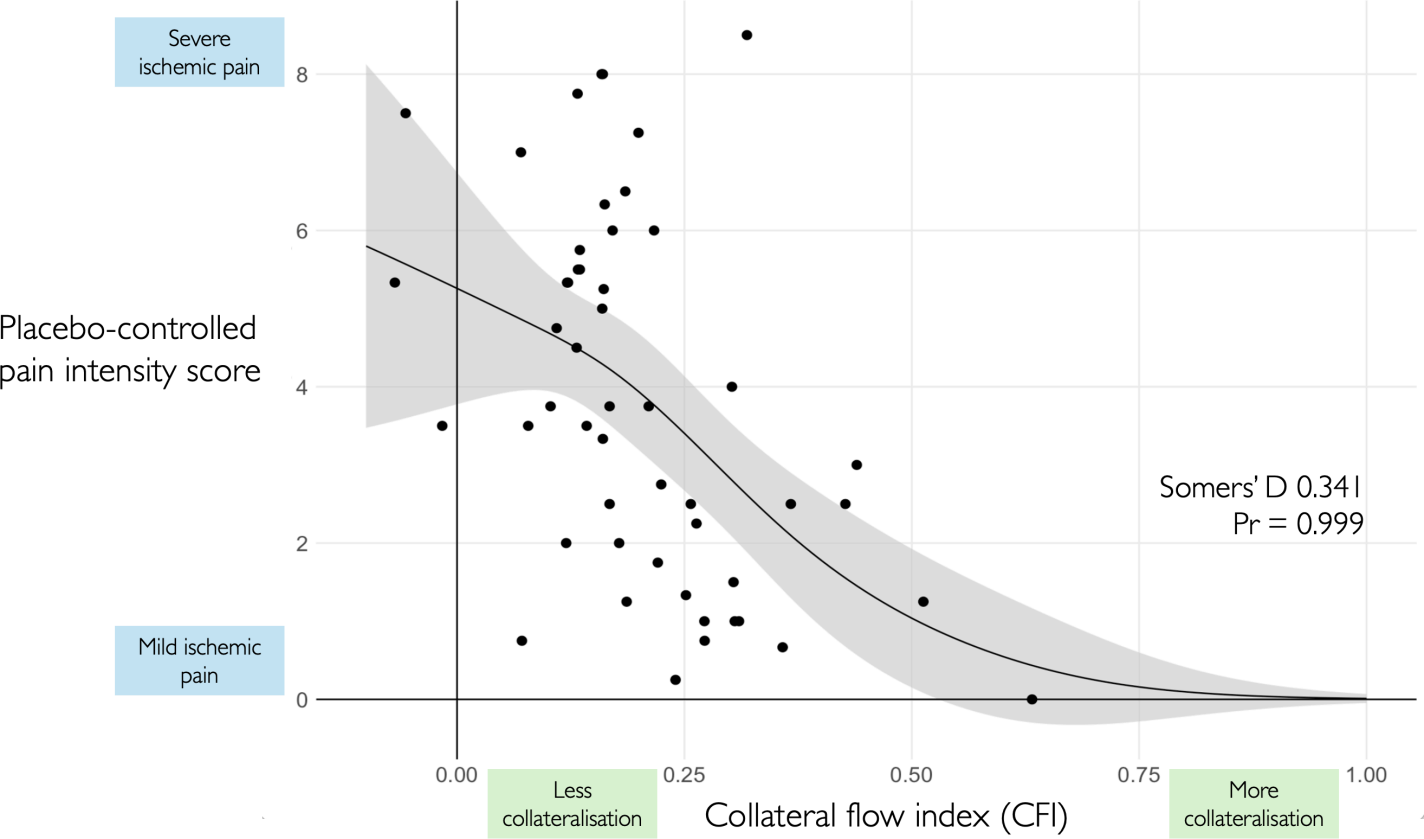
**Greater collateralization, as indicated by higher collateral flow index (CFI), was associated with significantly reduced (milder) placebo-controlled pain intensity scores (*Pr*>0=0.999).** Participants with less collateral blood flow were more likely to have severe pain with balloon occlusion.

There was strong evidence of an association between CFI and the placebo-controlled pain intensity score, indicating less ischemic pain in the presence of more collateralization (Somers’ D 0.341, *Pr*>0=0.999; Figure 5).

### Change in Pain Intensity Scores With Sequential Balloon Occlusion Episodes

The CFI was compared from the first to the fourth balloon inflation to test for the presence of progressive collateral recruitment as a possible mechanism for ischemic preconditioning. There was no progressive increase in CFI across the cohort (model-predicted mean CFI for the first inflation 0.19 [0.16–0.22] and fourth inflation 0.18 [0.16–0.21], *Pr*>0=0.032; Figure S3A).

Concordantly, there was no reduction in the pain intensity score between the first balloon inflation and the fourth balloon inflation (model-predicted mean pain intensity score for the first inflation 4.6 [3.6–5.7] and fourth inflation 5.1 [3.9–6.2], *Pr*>0=0.840; Figure S3B).

## Discussion

In this study of patients with single-vessel CAD and stable chest pain, there was little relationship between severity of ischemia and the daily burden of patient-reported symptoms. However, stenoses with greater flow limitation and perfusion abnormalities were associated with increased collateral flow, suggesting that the collateral circulation may adapt in the presence of chronic ischemia. The clinical significance of collateral flow was evaluated using a placebo-controlled pain intensity score, which revealed an inverse relationship: higher collateral flow was associated with reduced severity of ischemic chest pain. Finally, there was no evidence of acute ischemic preconditioning within this study.

### Collateralization as an Adaptive Response to Ischemia

Previous studies have identified anatomic severity of CAD as a principal determinant of collateralization, with other factors such as age, duration of angina, left ventricular hypertrophy, and systemic conditions such as anemia also playing a role.^19–23^ The unifying association that connects each of these predictive factors is their mechanistic link to supply-demand mismatch.

The presence of a trans-stenotic pressure gradient is a principal stimulus for arteriogenesis.^24^ Preclinical studies have shown that increased hemodynamic shear stress caused by larger pressure gradients between the unobstructed donor and stenosed recipient vessel promote changes in gene expression that upregulate angiogenic growth factors such as basic fibroblast growth factor and transforming growth factor β.^25,26^ Separately, coronary autoregulation with vasodilation of the microcirculation is a natural response that maintains anterograde perfusion through a stenosed vessel.^27^

Notably, FFR and iFR in both donor and recipient vessels are dependent on the CFI. This study assesses the relationship between collateral function and pressure gradients in the stenosed recipient vessel. Increases in collateral flow will increase the pressure distal to the coronary stenosis in the recipient vessel, increasing the distal pressure, FFR and iFR. Despite this interdependence, a highly significant relationship was seen between CFI and both FFR and iFR. Greater flow limitation in the diseased vessel was associated with a higher CFI. This relationship suggests that although the collateral circulation partially adapts to compensate for ischemia, this process does not fully resolve pressure gradients in severe CAD.

### Impact of CFI on Pain Intensity During Ischemia

In theory, a functional manifestation of collateralization may be a reduction of ischemic chest pain through improved perfusion of ischemic myocardium. Historically, the presence or absence of chest pain during coronary balloon occlusion served as one of the simplest, albeit crude, assessments of coronary collateral function.^28^

However, previous studies examining this phenomenon had several limitations. Simplistic dichotomization of angina as either present or absent is imprecise and restricts statistical power, as pain, like all biological variables, is continuous. Also, the assessment of chest pain during cardiac catheterization requires placebo-control because patients undergoing cardiac catheterization may experience symptoms for many reasons during the procedure beyond the specific manifestations of ischemia. Finally, blinding is particularly important. When patients are informed that blood flow is being restricted to the heart, this will naturally have a nocebo effect that manifests as perception of pain in some.

This study overcame these limitations by, for the first time, using a placebo-inflation comparator. It blinded the patient to remove the power of preconceived beliefs from their reported symptoms and served as a control for other sensations the patient may experience during cardiac catheterization. Additionally, an ordinal scale was used for pain intensity assessment during real and placebo balloon inflation episodes, giving maximal statistical power and granularity to the assessment of pain intensity.

This design showed that patients with greater collateralization had significantly reduced placebo-controlled pain intensity scores, confirming the hypothesis that the collateral circulation has clinical relevance in reducing the symptoms of ischemia.

These findings have direct clinical relevance. Physicians often question why some patients with extensive CAD and a high ischemic burden experience minimal symptoms. One hypothesis is that patients may subconsciously limit their activity to remain below the symptomatic threshold. However, these data suggest an alternative explanation: collateralization can reduce the association between severity of disease and symptom burden. Furthermore, in practice, part of the success of antianginal medical therapy may not solely be attributable to the pharmacological effects but also the ability to afford sufficient time for collateral flow to develop and mature.

The severity of ischemic chest pain is likely to be dependent on several factors. Aside from the role of collateralization, the intensity of ischemic chest pain may also vary depending on the duration of ischemia, volume of myocardium at risk, myocardial viability, autonomic nerve dysfunction, individual pain threshold, and psychological characteristics of the patient. When these factors are taken into consideration, it is perhaps less surprising that a direct and predictable relationship between ischemia and angina does not exist. This variability also underscores why the expectation of angina relief from revascularization is less reliable than previously assumed. Notably, although there was strong evidence of some of the associations identified in this study, the individual correlation coefficients (Somers’ D) were at best moderate, highlighting considerable variability between patients.

### Adaptation to Ischemia: Collateralization or Ischemic Preconditioning?

Ischemic preconditioning describes myocardial adaptation triggered by repeated short durations of ischemia and reperfusion. This phenomenon was first described in dogs, in which smaller infarct sizes were observed after preconditioning with 4 preceding 5-minute coronary occlusions.^29^ There was no difference in collateral flow between preconditioned and nonpreconditioned dogs; however, the quantity of collateral blood flow did predict the infarct size in the control arm. Subsequently, in humans, myocardial adaptation to repeated episodes of ischemia was attributed to both progressive collateral recruitment and separately to ischemic preconditioning, a biochemically driven phenomenon thought to be distinct from collateralization.^30,31^

Although previous studies have documented a progressive rise in CFI with repeated episodes of balloon occlusion, this was not observed in this cohort.^30^ Similarly, no progressive attenuation of pain intensity score was seen on repeated balloon occlusion episodes, in contrast to other observers.^30^ As a result, this study cannot draw conclusions about the mechanism of myocardial adaptation to ischemia, as no detectable adaptation was observed.

There are several possible explanations for these divergent results. First, the balloon occlusion episodes were shorter in this study than the 2- or 3-minute balloon occlusion episodes performed previously.^30,31^ Second, by design, participants with a broad spectrum of stenosis severity and burden of ischemia were enrolled in this study. These different substrates may respond differently to sequential balloon occlusion. Finally, this study was blinded with the incorporation of placebo control in a randomized order. In unblinded studies, a reduction in pain with subsequent episodes may be expected because the patient becomes psychologically conditioned to an anticipated sensation.

### Limitations

The assessment of the collateral circulation was based upon the continuous quantifiable hemodynamic metric of CFI. The angiography-derived Rentrop collateral grading system was not used because this is an ordinal classification that would reduce statistical power^32^ and does not account for the expected vasodilatation of patients with severe CAD. Use of CFI is also subject to less interobserver variability because it does not require interpretation of angiography images. Finally, most patients had no visible collateralization and presented with stable angina and a range of stenosis severities. Use of the more sensitive CFI permitted quantification of the lower collateral pressures typical of this population. It is possible that some of the patients in this study had impaired microcirculatory vasodilatation and severe CAD. The impact of microcirculatory dysfunction in patients with epicardial disease on CFI is unknown.

This study only included patients with single-vessel CAD. This retained the simplicity of the experimental design and allowed for precise assessment of how unobstructed donor arteries contribute to collateralization of a diseased vessel. The conclusions are therefore limited to a single-vessel disease population rather than a multivessel disease cohort, in which the observed associations are likely to be more complex and are the subject of an ongoing study (URL: https://www.clinicaltrials.gov; Unique identifier: NCT06400290).

A Combowire was placed in the distal vessel during the balloon occlusion protocol to ensure that resting flow conditions had returned to baseline, with resolution of reactive hyperemia, before the next episode began. This was to prevent any hemodynamic carryover between balloon occlusion episodes. Resolution of any pain after a balloon occlusion was required before continuation of the experimental protocol was allowed. Despite these efforts, some carryover of symptoms or hemodynamic disturbance from one episode to another remains a possibility.

Patients for this study were recruited from routine clinical PCI waiting lists. Selection bias may have impacted patient distributions because the symptom threshold required to refer patients for PCI may have been lower in patients with more severe disease.

Finally, the relatively small sample size will have increased the risk of a type II error.

### Conclusions

Development of the collateral circulation is an adaptive response to ischemia in patients with severe single-vessel stable CAD. This collateralization has clinical relevance in attenuating the severity of ischemic chest pain and may be partly responsible for the nonlinear relationship between stenosis severity, ischemia, and symptom burden in patients referred for PCI.

## Article Information

### Acknowledgments

ORBITA-STAR was an investigator-initiated trial sponsored by Imperial College London. The authors thank all patients and their families for their dedication and altruism, without which this research would not be possible. The authors acknowledge the support of the National Institute for Health and Care Research (NIHR) Clinical Research Network. They also thank the research and administrative teams at each of the 5 trial centers. Finally, the authors thank the National Health Service of the United Kingdom for encouraging and facilitating novel clinical research.

### Sources of Funding

Drs Rajkumar, Foley, and Ahmed-Jushuf are recipients of clinical research training fellowships from the Medical Research Council (MR/S021108/1, MR/V001620/1, and MR/W000520/1). Dr Chotai is recipient of a clinical research training fellowship from the National Institute for Health and Care Research (NIHR; 302493). Dr Al-Lamee is recipient of an intermediate research fellowship from the British Heart Foundation (FS/ICRF/22/26051). The authors acknowledge support from the NIHR Imperial Biomedical Research Centre and Imperial College British Heart Foundation Centre of Research Excellence.

### Disclosures

Dr Rajkumar reports consultancy fees from Philips and shares in Mycardium AI. Dr Foley reports speaker fees from Menarini and Philips. Dr Simader reports sponsorship from Servier Pharmaceuticals. Dr Sen reports speaker and consultancy fees from Philips, Medtronic, Recor, and AstraZeneca. Dr Petraco reports consultancy fees from Philips and Abbott. Dr Nijjer reports speaker fees from Philips Volcano, Pfizer, Bayer, AstraZeneca, Boehringer Ingelheim, and Amarin. Dr Howard and Dr Cole report shares in Mycardium AI. Dr Davies reports grants from Medtronic and Abbott; sponsorship from Vascular Perspectives, Boston Scientific, Medtronic, and Abbott; and speaker honoraria from AstraZeneca, Pfizer, Bristol Myers Squibb, and Novartis. Dr Kotecha reports honoraria from Bayer and Jansen. Dr Keeble reports advisory board for Abbott Vascular, Philips, and Abiomed and institutional research funding from Terumo and Abbott Vascular. Dr O’Kane reports speaker fees from Abbott Vascular, Biosensors, Boston Scientific, Heartflow, Medtronic, Philips, Shockwave, and Terumo. Dr Al-Lamee reports advisory board for Janssen Pharmaceuticals, Abbott, and Philips and speaker honoraria from Abbott, Philips, Medtronic, Servier, Omniprex, and Menarini.

### Supplemental Material

Supplemental Methods

Tables S1 and S2

Figures S1–S3

STROBE Checklist

## Supplementary Material
