# Supplementary material for: The Role of the Collateral Circulation in Stable Angina: An Invasive Placebo-Controlled Study
Source: Circulation. 2025 Oct 27;152(22):1541–51. doi: 10.1161/CIRCULATIONAHA.125.074687 (PMC12655870; doi:10.1161/CIRCULATIONAHA.125.074687)
Supplement: Supplementary file 1 [file cir-152-1541-s001.pdf]

# Supplemental Material

**The role of the collateral circulation in stable angina:**

**An invasive placebo-controlled study.**

Contents


**Supplemental Methods: Smartphone application description ........................ 3**

**Table S1: Inclusion Criteria ........................................................................ 4**

**Table S2: Exclusion Criteria ....................................................................... 4**

**Supplemental Figure S1: Smartphone App ..................................................... 5**

**Supplemental Figure S2: Consort Diagram ..................................................... 6**

**Supplemental Figure S3: Assessment for ischemic preconditioning ............. 7**

# Supplemental Methods: Smartphone application description

Symptom burden in ORBITA-STAR was assessed using a smartphone app. This was used to derive symptom frequency data for the primary endpoint. At enrolment, participants defined their symptom of angina in their own words. Participants then reported the number of episodes of this symptom for every day of the trial. The participants also pre-specified, at enrolment, two activities that currently triggered their symptom. The smartphone app asked participants to complete these two activities at least once weekly, and report whether they continued to trigger their symptom.

Full details regarding development and use of the application have previously published[1].

For the ORBITA-STAR trial, the smartphone application was only available in the English language. The smartphone application was intentionally designed to be very simple. An ethically and gender diverse patient focus group with lived experience of coronary artery disease assisted in the design of the smartphone application. Predominantly, participants were able to read, write and speak English. However, there was a minority of participants who could not read, write, or speak English. Most of these patients were assisted by a contact who was capable of translating. If participants did not have a contact who could offer this help, a blinded member of the research team arranged daily data entry using a translator service.

At enrolment, participants completed a training module, with a test component, which demonstrated and documented their ability to understand the app and input data.

Participants had 24/7 access to a blinded member of the trial team for any queries about the smartphone application.

Supplemental figure 1 contains screenshots from the ORBITA-STAR symptom application.

## Table S1: Inclusion Criteria

| Inclusion Criteria |
|---|
| Angina or angina equivalent symptoms |
| ≥70% stenosis of a single coronary artery on invasive coronary angiogram or CTCA |
| Referred for PCI |

*PCI = Percutaneous coronary intervention. CTCA: Computed tomography coronary angiography*

## Table S2: Exclusion Criteria

| Exclusion Criteria |
|---|
| Age younger than 18 years or older than 85 years |
| Recent acute coronary syndrome (< 6 months) |
| Multivessel coronary artery disease |
| Previous coronary artery bypass graft |
| Significant left main stem coronary disease |
| Chronic total occlusion in the target vessel |
| Contraindication to percutaneous coronary intervention or drug-eluting stent implantation |
| Contraindication to antiplatelet therapy |
| Contraindication to adenosine infusion |
| Severe valvular disease |
| Severe left ventricular systolic impairment |
| Severe respiratory disease |
| Life expectancy less than 2 years, pregnancy, unable to consent |

# Supplemental Figure S1: Smartphone App

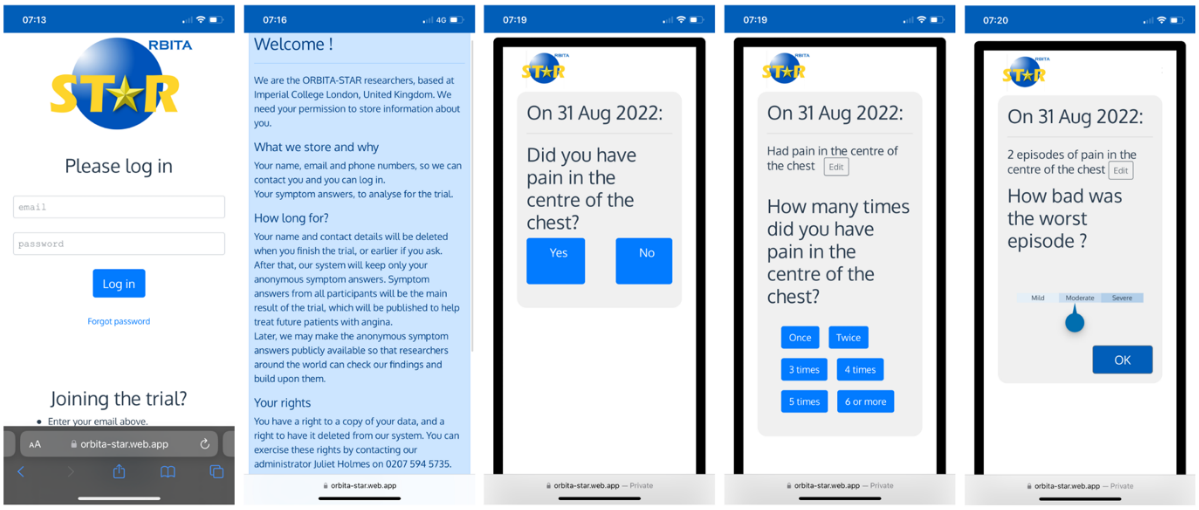


*Screenshots of the ORBITA-app. Participants used this app to record their daily symptoms of angina. Inputs were transferred to the research team instantaneously and stored in a secure server.*

Supplemental Figure S2: Consort Diagram

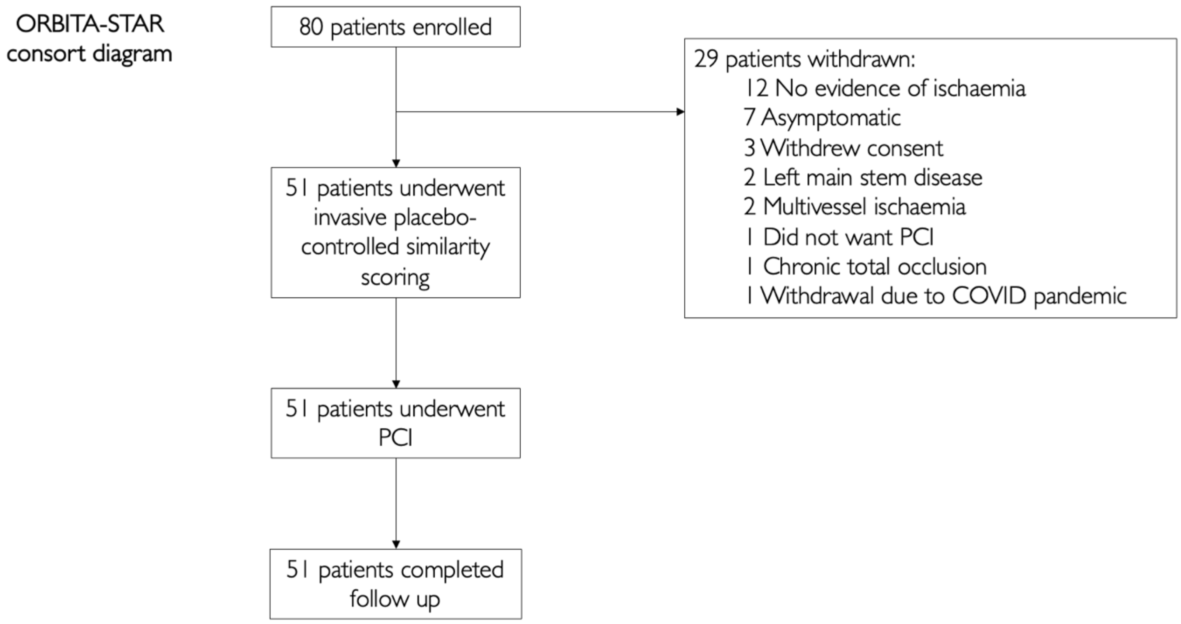


*Each withdrawn participant was allocated a singular reason for exclusion.*

## Supplemental Figure S3: Assessment for ischemic preconditioning

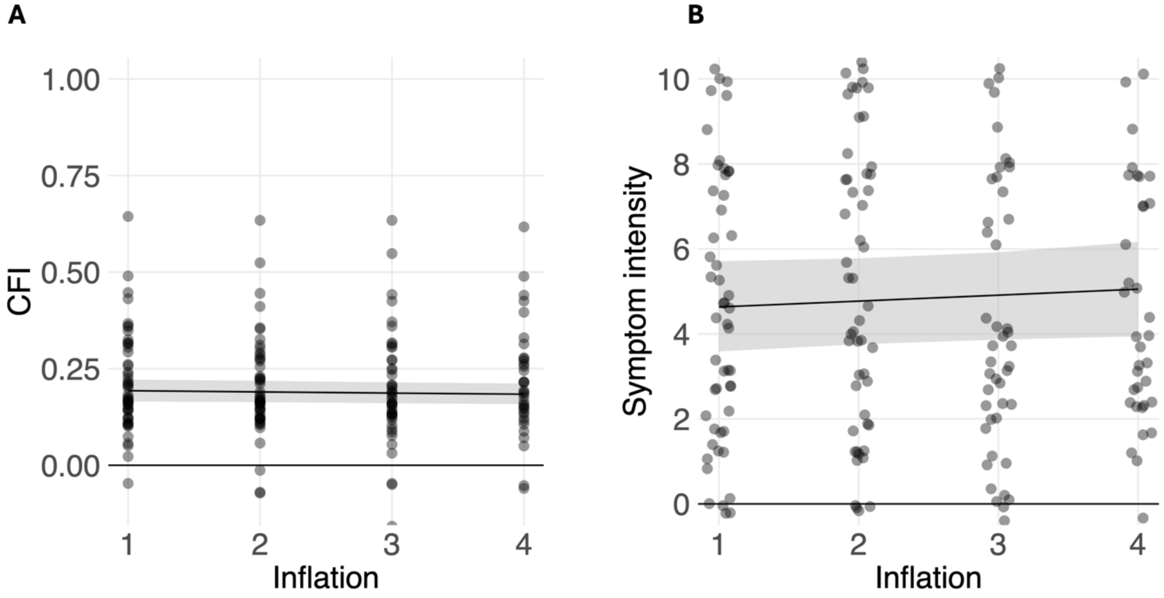


*Figure S3A: To assess for the presence of progressive collateral recruitment, CFI was compared over progressive balloon occlusion episodes. No significant increase in CFI was seen from the first balloon inflation to the fourth.*

*Figure S3B: To assess for the presence of ischemic pre-conditioning, the placebo-controlled pain intensity score was compared over progressive balloon inflation episodes. No significant reduction in pain intensity was observed from the first inflation to the fourth.*
